# Cirsilineol inhibits RANKL-induced osteoclast activity and ovariectomy-induced bone loss via NF-κb/ERK/p38 signaling pathways

**DOI:** 10.1186/s13020-024-00938-6

**Published:** 2024-05-14

**Authors:** Cong Wang, Rong Zeng, Yong Li, Rongxin He

**Affiliations:** 1 Department of Orthopedic Surgery, The Second Affiliated Hospital, School of Medicine, Zhejiang University, Hangzhou, Zhejiang People’s Republic of China; 2 Orthopedics Research Institute of Zhejiang University, Hangzhou, Zhejiang People’s Republic of China; 3 Key Laboratory of Motor System Disease Research and Precision Therapy of Zhejiang Province, Hangzhou, Zhejiang People’s Republic of China; 4 Clinical Research Center of Motor System Disease of Zhejiang Province, Hangzhou, Zhejiang People’s Republic of China; 5 Pain Management, YiChun People’s Hospital, Yichun, Jiangxi People’s Republic of China; 6 Department of Orthopedics, Qingtian People’s Hospital, Lishui, Zhejiang People’s Republic of China

**Keywords:** Cirsilineol, RANKL, Osteoclast, NK-κb, ERK, p38

## Abstract

**Background:**

Postmenopausal osteoporosis is a chronic metabolic bone disease caused by excessive osteoclast formation and function. Targeting osteoclast differentiation and activity can modulate bone resorption and alleviate osteoporosis. Cirsilineol, an active constituent of Vestita Wall, has shown numerous biological activities and has been used to treat many metabolic diseases. However, whether cirsilineol inhibits osteoclast activity and prevents postmenopausal osteoporosis still remain unknown.

**Materials and methods:**

Primary bone marrow macrophages (BMMs) and RAW264.7 cells were used. Osteoclast activity was measured by TRAP staining, F-actin staining, and bone resorption assay after BMMs were treated with cirsilineol at concentrations of 0, 1, 2.5 and 5 µM. RT-PCR and western blotting were performed to evaluate the expression of osteoclast-related genes. In addition, female C57BL/6 mice underwent OVX surgery and were treated with cirsilineol (20 mg/kg) to demonstrate the effect of cirsilineol on osteoporosis.

**Results:**

Cirsilineol significantly inhibited receptor activator of nuclear factor-kappa B ligand (RANKL)-induced osteoclast differentiation in a concentration- and time-dependent manner, respectively. Additionally, cirsilineol inhibited F-actin ring formation, thus reducing the activation of bone resorption ability. Cirsilineol suppressed the expression of osteoclast-related genes and proteins via blocking nuclear factor (NF)-κb, ERK, and p38 signaling cascades. More importantly, cirsilineol treatment in mice with osteoporosis alleviated osteoclasts hyperactivation and bone mass loss caused by estrogen depletion.

**Conclusion:**

In this study, the protective effect of cirsilineol on osteoporosis has been investigated for the first time. In conclusion, our findings prove the inhibitory effect of cirsilineol on osteoclast activity via NF-κb/ERK/p38 signaling pathways and strongapplication of cirsilineol can be proposed as a potential therapeutic strategy.

**Supplementary Information:**

The online version contains supplementary material available at 10.1186/s13020-024-00938-6.

## Introduction

Postmenopausal osteoporosis is a fairly common chronic disease characterized by abnormal bone metabolism and ultimately leads to decreased bone volume [1]. The decoupling of bone formation and bone resorption, which means the overactivation of osteoclasts and the silencing of osteoblasts, is the most common culprit in osteoporosis [2]. Osteoporosis usually has a poor prognosis and is accompanied by many complications, including systemic pain, blood vessel damage, nerve inflammation and a more increased risk of fractures [3–5]. Pathological fractures combine osteoporosis influence the activity of daily living of patients and can be life-threatening in severe cases [6].

Osteoclasts are multinucleated giant cells formed by the fusion of monocytes or macrophages derived from myeloid progenitor cells in bone marrow and are considered to be the sole regulator in osteolysis. Osteoclast precursors promote the expression of RANK on the cell surface in response to macrophage colony-stimulating factor (M-CSF) stimulation [7], making it more sensitive to RANKL signals. Binding of RANKL and RANK initiates the downstream signaling cascadeS including protein kinases (MAPKs), and NF-κb and thus triggers the activation of downstream osteoclast-related genes, leading to osteoclast differentiation [8, 9].

Excessive bone resorption by osteoclasts has been implicated in osteoporosis-induced bone mass loss, and the formation of F-actin ring makes a very positive contribution in this process [10]. F-actin ring, also known as sealing zone, forms during osteoclasts polarization and morphological changes, surrounding the resorption cavity and supporting the degradation of ECM by promoting environmental acidification [11–13]. F-actin ring formation is crucial for osteolytic activity.

Sustained inflammatory microenvironment and elevated inflammatory mediators are highly correlated with decreased bone mass and bone density in many patients with chronic inflammatory diseases [14, 15]. As previously reported, increased levels of inflammatory markers trigger the RANKL secretion from stromal cells, activate the RANKL/RANK signaling pathways in osteoclasts and affect bone metabolism [16, 17]. For the moment, inhibition of inflammation is an effective method to retard bone loss [18].

Anti-osteoporosis drugs mainly include basic supplements, bone resorption inhibitors and bone formation promoters [19, 20]. Clinically, inhibition of osteoclastic bone resorption is the main therapeutic strategy [21]. However, some of these drugs neither improve symptoms nor prevent side effects. Most drugs even show severe side effects ranging from gastro-intestinal tract bad effect to hepatorenal damage [22, 23].

Cirsilineol (4ʹ,5-dihydroxy-3ʹ,6,7-trimethoxyflavone), a kind of flavonoids bioactive compound isolated from vestita Wall, has been previously reported to possess numerous biological activities including anti-inflammatory [24], anti-tumor [25, 26], anti-plasmodial [27] and gastroprotective effect [28]. For local inflammation caused by Allergic Rhinitis, cirsilineol has shown a protective effect on immune imbalance by reducing inflammatory factor levels [29]. Additionally, cirsilineol has been reported to show considerable antibacterial activity against Helicobacter pylori [30]. Besides, a previous study has also shown that cirsilineol displayed excellent antioxidant and antidiabetic potential [31]. Given the diverse biological activities of cirsilineol, it has merged as a promising and safe approach to treat various diseases.

Considering the further evidence for links between oxidative stress, inflammatory processes and osteoclast activation, we investigated for the first time whether cirsilineol exerted protective effect on osteoporosis. Our studies suggest that cirsilineol suppresses RANKL-induced osteoclast differentiation and ameliorates osteoporosis-induced bone mass loss in vivo.

## Materials and methods

### Reagents

**Fig. 1:**
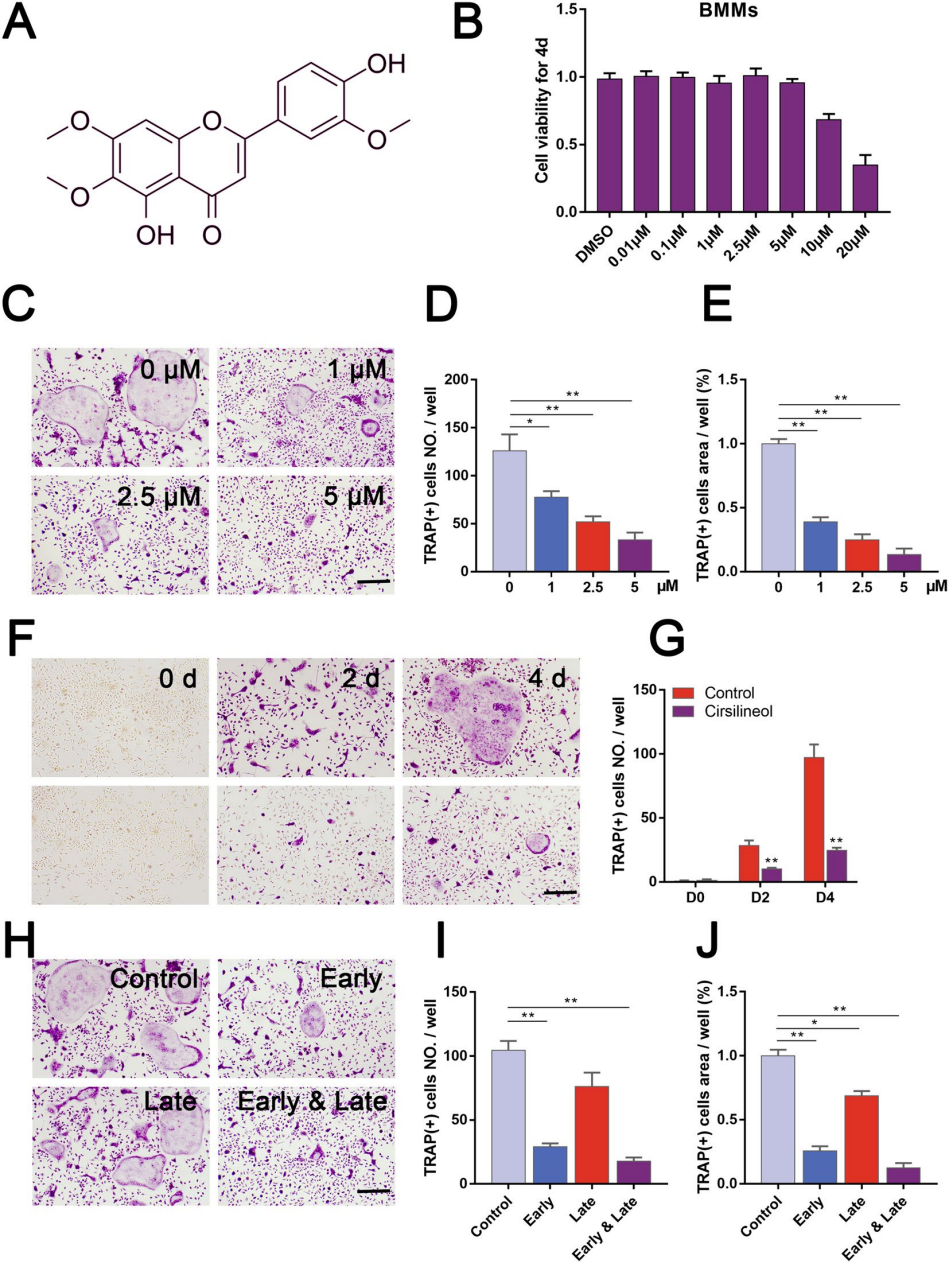
Inhibition of osteoclast differentiation by cirsilineol in vitro. **A** Chemical structure of cirsilineol. **B** Cirsilineol showed no toxicity to BMMs, until the concentration reaches 20 μM (n = 4). **C**–**G** Cirsilineol inhibited RANKL-induced osteoclast differentiation in a concentration- and time-dependent manner (n = 3). **H**–**J** The number and size of TRAP-positive osteoclasts both reduced in the early and late stage, and the inhibitory effect was more profound in the early stage (n = 3). Scale bar = 500 μm, *P < 0.05, **P < 0.01 vs. the control group

Cirsilineol (purity ≥ 97%; Fig. 1A) was purchased from MedChemExpress (MCE) Co. (New Jersey, America) and dissolved in dimethyl sulfoxide (DMSO) to make a solution of various concentrations. α-MEM and fetal bovine serum were purchased from Thermo Fisher Scientific (Scoresby, Vic, Australia). Recombinant human RANKL and M-CSF were purchased from Novoprotein Scientific Inc. (Shanghai, China). Rabbit-derived primary antibodies against p-ERK, ERK, p-JNK, JNK, p-p38, p38, p-p65, p65, p-ikβα, ikβα, p-akt, akt, NFATc1/NFAT2, c-Fos, RANK and GAPDH were all purchased form Cell Signaling Technology (Danvers, MA, USA). Second antibodies and CCK8 kits were acquired from Beyotime Biotechnology Co. (Shanghai, China). Tartrate-resistant acid phosphatase (TRAP) staining components and all other reagents were obtained from Sigma-Aldrich (St. Louis, MO, USA). All animals used for experiments were purchased from SLAC Laboratory Animal Co. (Shanghai, China).

### Cell isolation and cell viability assay

Long bones of both extremities were separated from 8-week-old C57BL/6 mice. By flushing the bone medullary cavity, macrophage precursors were obtained and then differentiated into macrophages with 50 ng/ml M-CSF stimulation for 5 days. All adherent cells were obtained for CCK8 assay and osteoclast differentiation.

To verify the cytotoxicity of cirsilineol, BMMs were seeded into 96-well plates and then cultured with different dosages of cirsilineol (0, 0.01, 0.1, 1, 2.5, 5, 10, 20 µM) for 1 day, 2 days and 4 days, respectively. In addition, MC3T3-E1 cells were also seeded into 96-well plates and cultured with different concentrations of cirsilineol (0, 0.5, 1, 2.5, 5, 10, 20, 50, 100 μM) for another 4 days. 10 μL CCK8 solution was added into each well and the change of cell viability was observed with CCK8 colorimeter using the ELX808 absorbance microplate reader (BioTek, Winooski, VT, USA).

### TRAP staining assay

To determine the inhibition of cirsilineol on osteoclast formation, BMMs were seeded into 24-well plates at a density of 2 × 10^4^ cells/well and cultured with osteoclastic medium containing 30 ng/ml M-CSF and 100 ng/ml RANKL for 4 days. During osteoclast differentiation, cells were also treated with different dosages of cirsilineol (0, 1, 2.5, 5 µM). After that, all cells were fixed with 4% paraformaldehyde and performed with TRAP staining assay. Mature osteoclasts meet the criteria of containing three or more nuclei. After the TRAP staining images were taken, the number and size of osteoclasts were quantitated compared to control.

### F-actin ring staining and bone resorption assay

Mature osteoclasts were incubated with indicated dosages of cirsilineol during RANKL stimulation. Then cells were washed with PBS and fixed in 4% paraformaldehyde for 30 min. After cultured with rhodamine-phalloidin for 1 h, all cells were gently washed for three times, stained with DAPI for 5 min, and photographed by confocal microscopy.

To investigate the inhibitory effect of cirsilineol on bone resorption ability, BMMs were plated into 12-well plates at a density of 2 × 10^5^ cells/well, and cultured with complete α-MEM containing 50 ng/ml M-CSF overnight. The cells were then stimulated with 100 ng/ml RANKL for 5 days until mature osteoclasts formed. Afterwards, all cells were thoroughly transplanted onto bone discs and treated with 5 μM cirsilineol for another 4 days. The resorption pits were photographed by the Hitachi (Chiyoda, Tokyo, Japan) S-3700N scanning electron microscope.

### Quantitative real-time PCR

In order to identify the inhibitory effect of cirsilineol on osteoclast formation and function in gene levels, BMMs were seeded into 12-well plates and cultured with or without indicated dosages of cirsilineol (0, 1, 2.5, 5 μM). Besides, MC3T3-E1 cells were also seeded into 12-well plates and then cultured with differnet concentrations of cirsilineol (0, 5, 10 μM). Total RNA was collected using Trizol reagent (Takara, Dalian, China) and transcribed back into cDNA, which used for quantitative real-time PCR (RT-PCR). The expression of osteoclast-specific gene was normalized to the level of GAPDH, and the experiments were repeated for three times. The primer sequences for rt-PCR are as follows: GAPDH (Forward: 5′-ACCACAGTCCATGCCATCAC-3′; Reverse: 3′-TCCACCACCCTGTTGCTGTA-5′), NFATc1 (forward: 5′-CCGTTGCTTCCAGAAAATAACA-3′; reverse: 3′-TGTGGGATGTGAACTCGGAA-5′), TRAP (forward: 5′-CACTCCCACCCTGAGATTTGT-3′; reverse: 3′-CCCCAGAGACATGATGAAGTCA-5′); V-ATPase-a3 (forward: 5′-GCCTCAGGGGAAGGCCAGATCG-3; reverse: 3′-GGCCACCTCTTCACTCCGGAA-5′), cathepsin K, (forward: 5′-CTTCCAATACGTGCAGCAGA-3′; reverse: 3′-TCTTCAGGGCTTTCTCGTTC-5′), DC-STAMP (forward: 5′-AAAACCCTTGGGCTGTTCTT-3′; reverse: 3′-AATCATGGACGACTCCTTGG-5′), MMP-9 (forward: 5′-CAAAGACCTGAAAACCTCCAA-3′; reverse: 3′-GGTACAAGTATGCCTCTGCCA-5′), Tnf-α (forward: 5′-AAGCCTGTAGCCCACGTCGTA-3′; 3′-AGGTACAACCCATCGGCTGG-5′), IL-1β (forward: 5′-AAAAAAGCCTCGTGCTGTCG-3’; reverse: 3’-GTCGTTGCTTGGTTCTCCTTG-5’), IL-6 (forward: 5′-TCCATCCAGTTG CCTTCTTG-3’; reverse: 3’-TTCCACGATTTCCCAGAGAAC-5’), Collagen I (forward: 5′-AGAGCATGACCGATGGATTC-3′; reverse: 3′-CCTTCTTGAGGTTGCCAGTC-5′), Runx2 (forward: 5′-AAGTGCGGTGCAAACTTTCT-3′; reverse: 3’-AAGTGCGGTGCAAACTTTCT-5′).

### Western blotting

Total cell lysate was collected and performed with high-speed centrifugation at 14,000 rpm for 15 min. All proteins were harvested from the supernatant of cell lysate. The obtained proteins were separated via SDS-PAGE electrophoresis and then transferred onto PVDF membranes for 2 h. The PVDF membranes were then blocked with 10% milk and incubated with primary antibodies overnight at 4 ℃. After washed with Tris-buffered saline three times, all samples were cultured with second antibodies for another 2 h at 4 ℃. Relative protein expression levels were detected using the Bio-Rad XRS chemiluminescence detection system (Hercules, CA, USA) and quantitated with the ImageJ software.

### Alizarin red S (ARS) and Alkaline phosphatase (ALP) activity assay

To investigate whether cirsilineol affected bone formation and osteoblasts differentiation, MC3T3-E1 cells were used for further examination. Specifically, MC3T3-E1 cells (2 × 10^5^ cells/well) were seeded into 6-well plates and then treated with different concentration of cirsilineol (0, 10, 20 μM) in osteogenic medium. Three days after osteogenic induction, MC3T3-E1 cells were fixed with 10% formaldehyde and then cultured with ALP staining kits. Another two weeks after osteogenic induction, MC3T3-E1 cells were incubated with 0.1% alizarin red dye for 30 min. After that, all plates were photographed using a high‐resolution microscope (Leica, Germany).

### Establishment of osteoporosis mice model and bone histologic analysis

To verify whether cirsilineol exerted protective effect on osteoporosis-induced bone mass loss, ovariectomy mice models were established. Thirty 8-week-old female C57BL/6 mice were purchased and then randomly divided into six different groups: sham-surgery group, OVX group, OVX + cirsilineol (10 mg/kg) group, OVX + cirsilineol (20 mg/kg) group, OVX + cirsilineol (30 mg/kg) group and OVX + Zoledronate (0.1 mg/kg). One week after surgery, the cirsilineol-treated group mice were intraperitoneally injected different dosage of cirsilineol every 2 d. Equal volume of placebo was injected into each mouse in the sham-surgery group and OVX group. Four weeks later, all mice were euthanized by an overdose of anesthetic. Bilateral femurs were separated, fixed with 4% paraformaldehyde solution for 1 day, and then evaluated by microcomputed tomography to determine the appropriate dosage of cirsilineol. After decalcified with 10% EDTA for a month, long bones were cut into 4-μm sections and all sections were performed with histomorphology staining.

### Statistical analysis

All experimental results are displayed as mean ± SD. Statistical difference was presented with Student's t-test and One-way ANOVA. P < 0.05 proved to be statistically significant.

## Results

### Cirsilineol inhibits RANKL-induced osteoclast formation, but shows no effect on osteoblast differentiation

Cell viability assay was performed to verify the toxic effect of cirsilineol on BMMs and MC3T3-E1 cells. After cirsilineol-treated BMMs and MC3T3-E1 cells were mixed with CCK-8 solution for 4 h, signal intensity was measured. The results demonstrated that the indicated concentrations of cirsilineol (0, 0.01, 0.1, 1, 2.5, 5 μM) had no obvious toxic effect after co-cultured with BMMs for 1 day, 2 days and 4 days, respectively (Fig. 1B). Besides, the results also showed that cirsilineol had no significant toxic effect on the cell viability of MC3T3-E1 cells, until cirsilineol was administrated at a concentration of 50 μM (Additional file 1: Fig. S1B).

Cirsilineol suppressed RANKL-induced osteoclast differentiation in concentration-dependent manner and time-dependent manner (Fig. 1C–G). Meanwhile, which stage did cirsilineol affect osteoclast formation was also been explored, including early stage (0–2 d), late stage (2–4 d) and all stage (0–4 d). The results showed that the number and size of TRAP staining positive cells both reduced in the early and late stage, and the early stage was more obviously inhibited (Fig. 1H–J). All data demonstrated that cirsilineol remarkably suppressed RANKL-induced osteoclast differentiation.

To better understand the effects of cirsilineol on bone formation, MC3T3-E1 cells were cultured with indicated concentrations of cirsilineol (0, 10, 20 μM) under osteogenesis-induced condition. Three days or fourth days after osteogenic induction, all cells were performed with ARS and ALP staining. Both imaging results and statistical results proved that cirsilineol had no obvious effects on osteoblasts differentiation (Additional file 1: Fig. S2A–C). Beyond that, there were also no significant differences in the expression of osteogenesis-related genes (Collagen I and Runx2) after cirsilineol treatment, either at mRNA or protein levels (Additional file 1: Fig. S2D, E).

### Cirsilineol inhibits F-actin ring formation and bone resorption ability

**Fig. 2:**
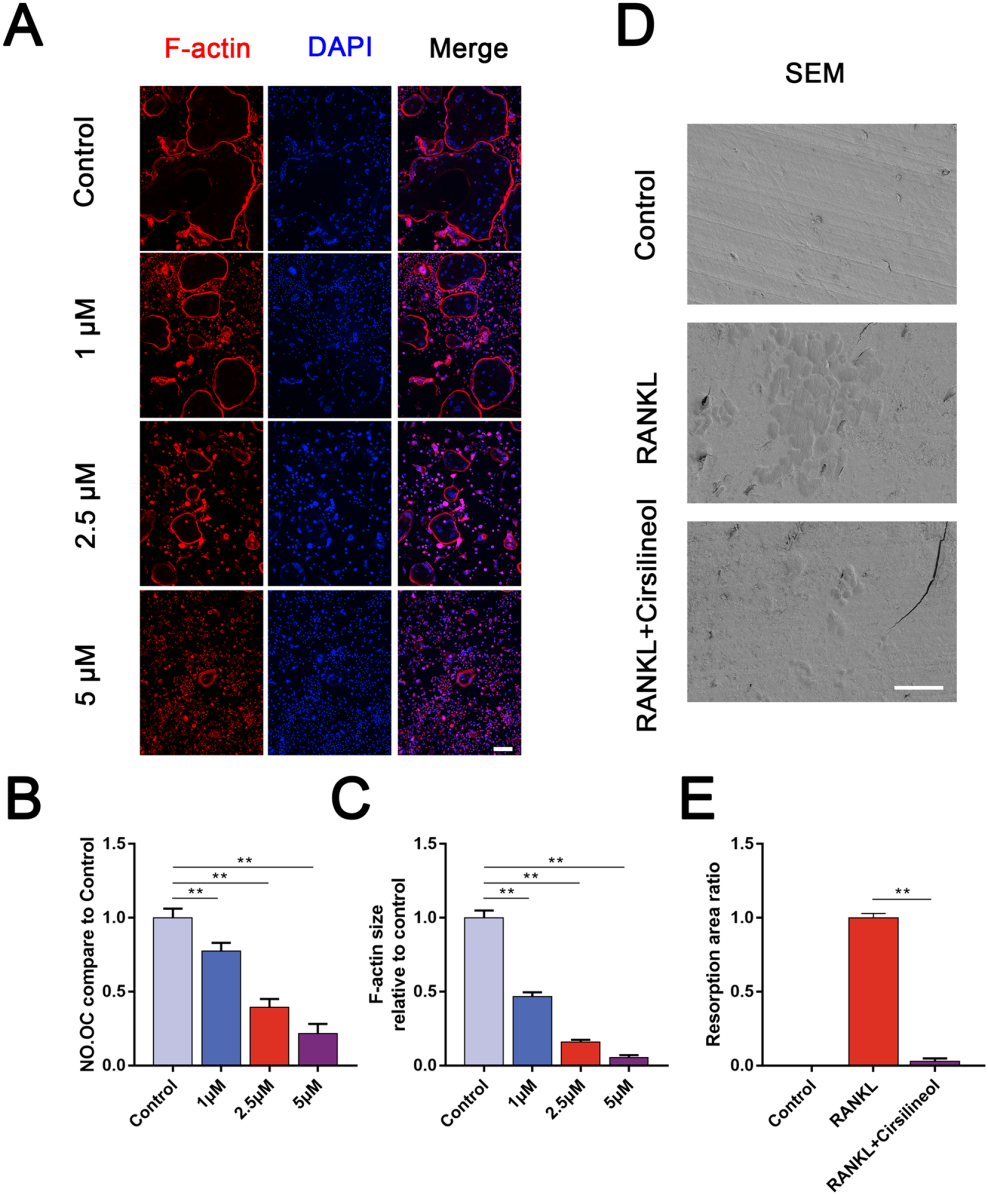
Cirsilineol inhibits bone resorption ability of mature osteoclast. **A**–**C** BMMs were differentiated into mature osteoclast for 4 days, and differentiated cells were treated with different concentrations of cirsilineol for another 2 days. The number of osteoclasts and the size of F-actin rings significantly reduced upon cirsilineol stimulation (n = 3). Scale bar = 50 μm. **D**, **E** Mature osteoclasts were seeded into bone slices and cultured with or without cirsilineol for 4 days. The osteolytic ability of osteoclasts was potently inhibited (n = 3). Scale bar = 50 μm.*P < 0.05, **P < 0.01 vs. the control group

In order to determine the inhibitory effect of cirsilineol on F-actin ring formation, BMMs were differentiated into mature osteoclasts with RANKL stimulation. Then mature osteoclasts were transplanted onto slides and incubated with different dosages of cirsilineol (0, 1, 2.5, 5 μM). After performed with rhodamine-phalloidin staining, the number and area of F-actin ring were photographed and analyzed. The results showed that the actin ring formation was most obviously inhibited with 5 μM cirsilineol treatment (Fig. 2A–C).

The degradation and absorption of bone matrix is the most important ability of mature osteoclasts and also reflects osteoclast vitality. Hence, we tested whether cirsilineol suppressed osteolytic ability of osteoclasts. And the results verified that the bone resorption area remarkably decreased after treated with cirsilineol (Fig. 2D, E).

### Cirsilineol inhibits osteoclast-specific genes expression and impairs NFATc1/c-Fos production

**Fig. 3:**
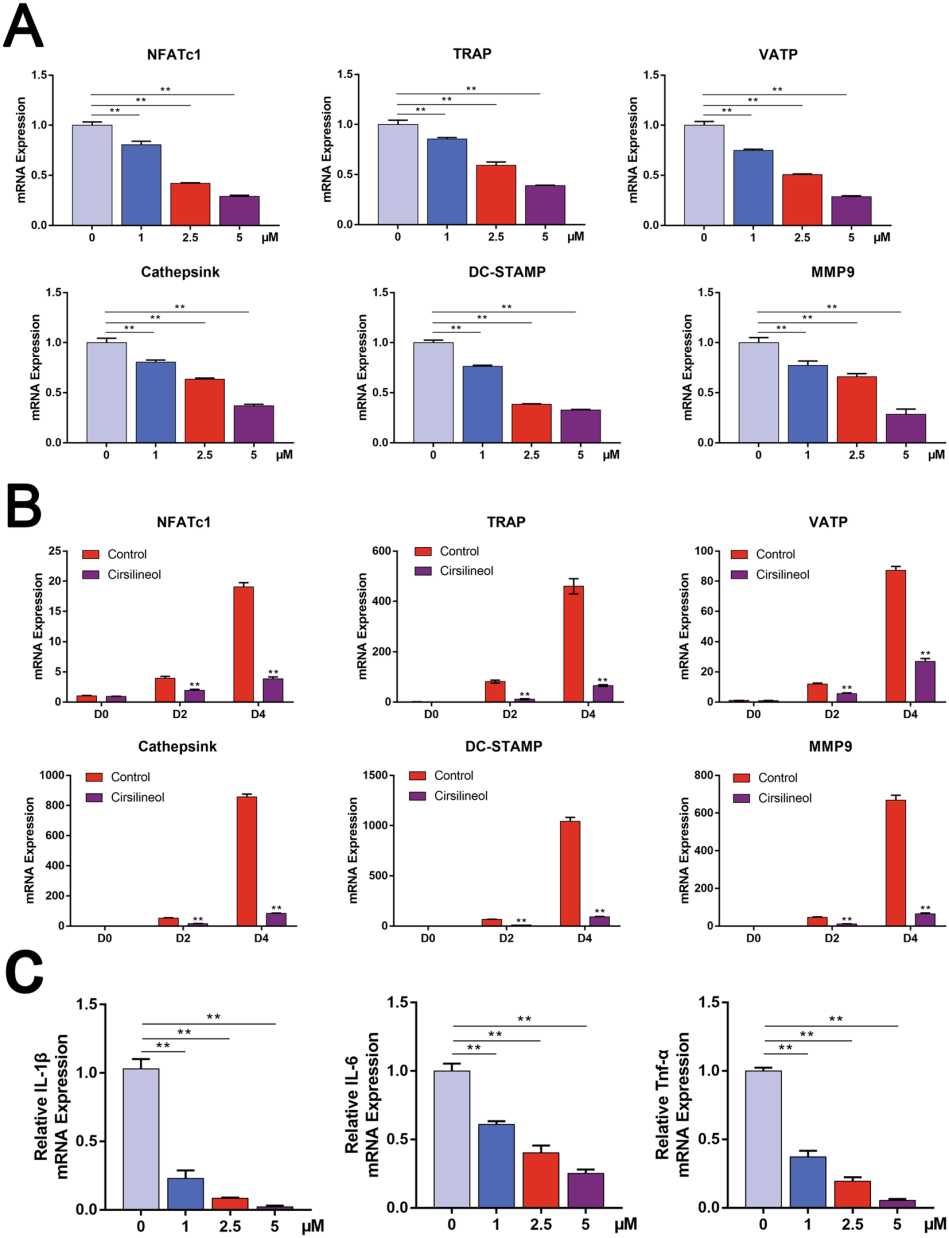
Cirsilineol inhibits osteoclast-specific genes expression at the transcriptional level. **A** BMMs were cultured with different concentrations of cirsilineol for 4 days, and the expression of osteoclast-specific genes including TRAP, DC-STAMP, CTSK, V-ATPase a3 and MMP-9 was significantly suppressed (n = 3). **B** BMMs were cultured with or without cirsilineol for 4 days, and the results showed that cirsilineol inhibited osteoclast-specific genes expression in a time-dependent manner (n = 3). **C** Cirsilineol inhibited the expression of inflammatory cytokines including IL-6, IL-1β and Tnf-α in a dose-dependent manner. *P < 0.05, **P < 0.01 vs. the control group

To investigate whether cirsilineol inhibits osteoclast formation and function in mRNA levels, numerous osteoclast-specific genes expression including NFATc1, TRAP, V-ATPase-a3, Cathepsink, DC-STAMP and MMP9 was detected with RT-PCR. The results confirmed that cirsilineol significantly suppressed the expression of osteoclast-related genes in a dosage- and time-dependent manner (Fig. 3A, B).

Postmenopausal sex steroid deficiency alters the production of several inflammatory cytokines, and in particular the cytokines IL-6, IL-1β and Tnf-α are regarded as the potent cytokines that promote bone resorption via activation of RANKL/RANK axis, thereby enhancing osteoclast activity [32–34]. In our study, we treated BMMs with different concentrations of cirsilineol (0, 1, 2.5, 5 μM) during osteoclastic induction. All results verified that cirsilineol inhibited the expression of IL-6, IL-1β and Tnf-α in a dose-dependent manner (Fig. 3C).

**Fig. 4:**
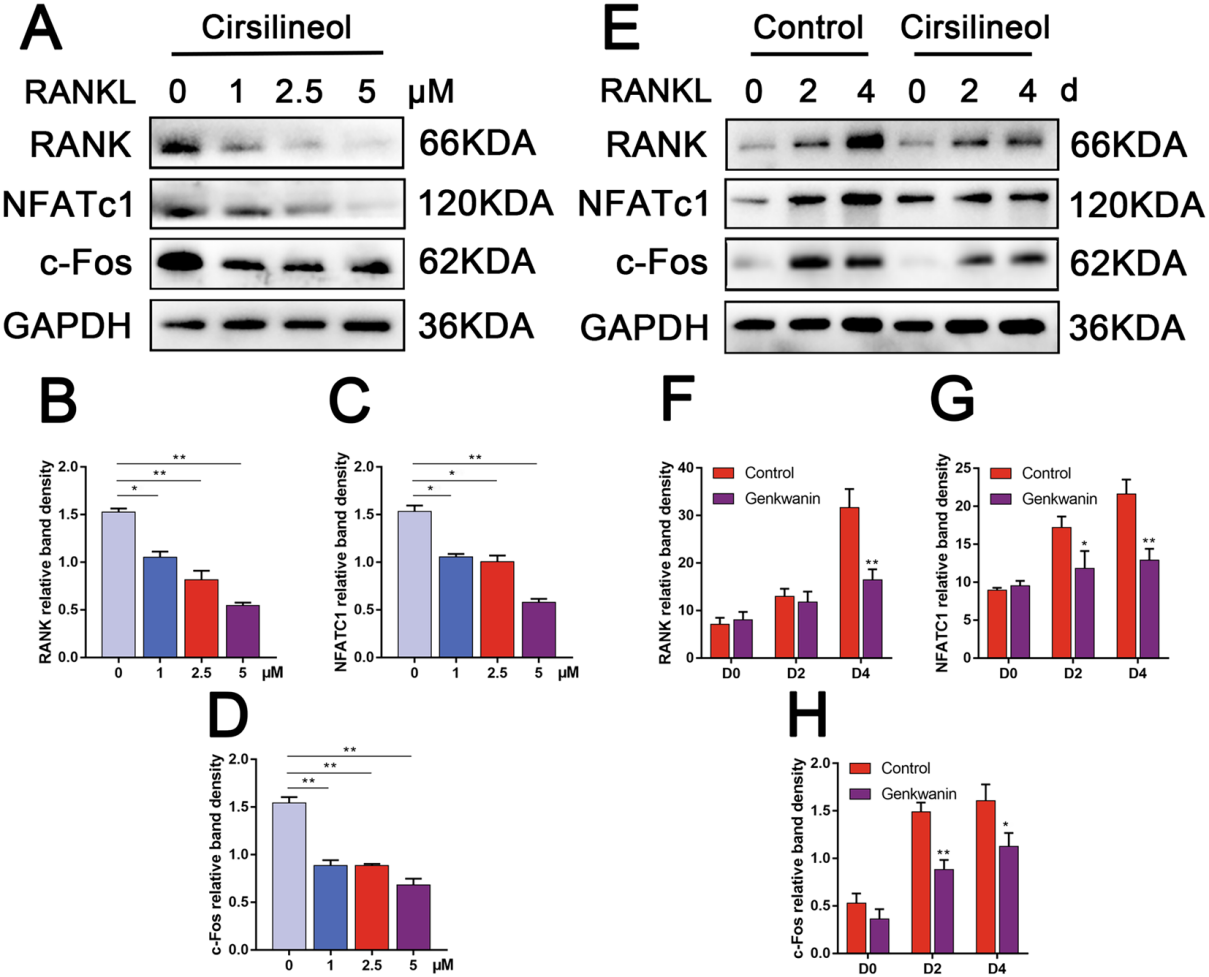
Cirsilineol inhibits osteoclast-specific genes expression at the translational level. **A**–**D** With the increase of cirsilineol’s concentration, the expression levels of RANK, NFATc1 and c-Fos were on a downward trajectory (n = 3). **E**–**H** BMMs were seeded into 12-well plates and incubated with or without 5 μM cirsilineol for 4 days. The expression levels of RANK, NFATc1 and c-Fos increased over time and were suppressed with cirsilineol treatment (n = 3). *P < 0.05, **P < 0.01 vs. the control group

M-CSF acts as a crucial regulator in promoting RANK expression during osteoclastogenesis [7]. Activation of RANKL/RANK signal initiates the downstream signaling pathways and regulates NFATc1/c-Fos inductions, which is involved in mediating actin re-organization and cell differentiation of osteoclast precursors [35]. In this study, we also found that the inhibitory effect of cirsilineol on the protein expression of RANK, NFATc1 and c-Fos becomes more pronounced as the concentration increases (Fig. 4A–D). Meanwhile, cirsilineol also inhibited the above protein expression in a time-dependent manner (Fig. 4E–H).

### Cirsilineol blocks RANKL-activated NF-κb/ERK/p38 signaling pathways

**Fig. 5:**
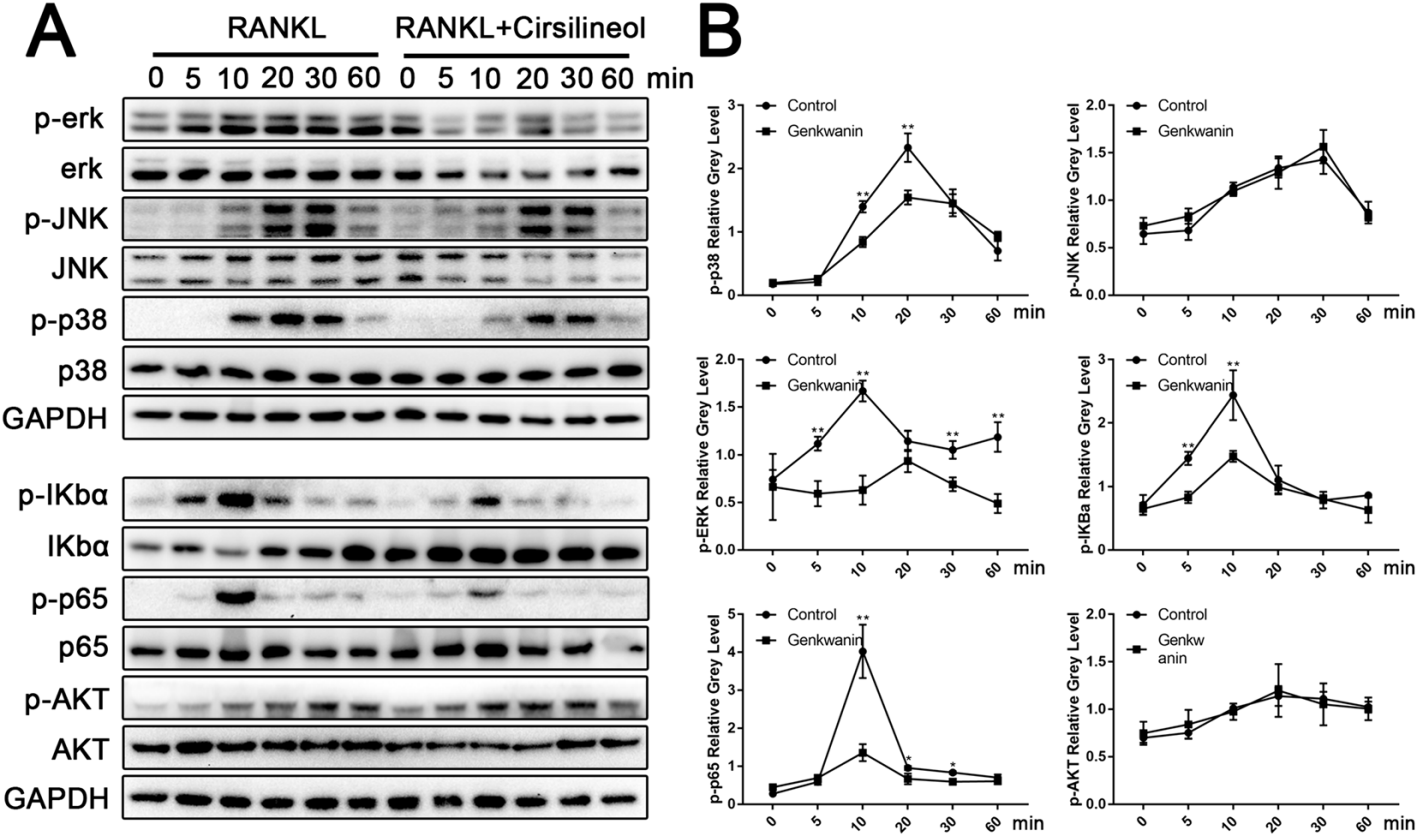
Cirsilineol blocks RANKL-induced NF-κb/ERK/p38 signaling pathways activation. **A**, **B** To investigate the mechanisms in which cirsilineol inhibits osteoclast formation and activation, RAW264.7 cells were seeded into 6-well plates and cultured with or without cirsilineol for 6 h. Then all cells were stimulated with 100 ng/ml RANKL for 0, 5, 10, 20, 30, 60 min, respectively. The results demonstrated that cirsilineol inhibited the activation of NF-κb/ERK/p38 signaling cascades (n = 3). *P < 0.05, **P < 0.01 vs. the control group

In order to elucidate the exact molecular mechanism by which cirsilineol affected the activation of downstream signaling pathways, RAW264.7 cells were seeded into 6-well plates at a density of 8 × 10^5^ cells/well. After pretreated with or without 5 μM cirsilineol for 6 h, all cells were stimulated with 100 ng/ml RANKL for 0, 5, 10, 20, 30, 60 min, respectively. Then total proteins were isolated and used for detecting the phosphorylation levels of MAPKs, AKT and NF-κb signaling cascades. The protein levels of p-ERK and p38 in cirsilineol -treated group obviously decreased while compared to control group. Nevertheless, the activation of JNK, which are also known to be part of the MAPK family, seem to make no significant difference (Fig. 5A, B).

NF-κb signaling cascade also plays an essential role in osteoclast differentiation and function [9]. However, the phosphorylation levels of p65 significantly reduced in the presence of 5 μM cirsilineol. In the meantime, the activation of the upstream IκBα pathway was also inhibited after cirsilineol treatment, which resulted in the decrease of p-p65 protein expression. Akt has been regarded as a master regulator in osteoclast differentiation and survival. In our study, we detected the phosphorylation of AKT, and the result proved that cirsilineol had no effect on p-AKT level (Fig. 5A, B).

### Cirsilineol prevents OVX-induced bone mass loss and inhibits osteoclast activity in mice

**Fig. 6:**
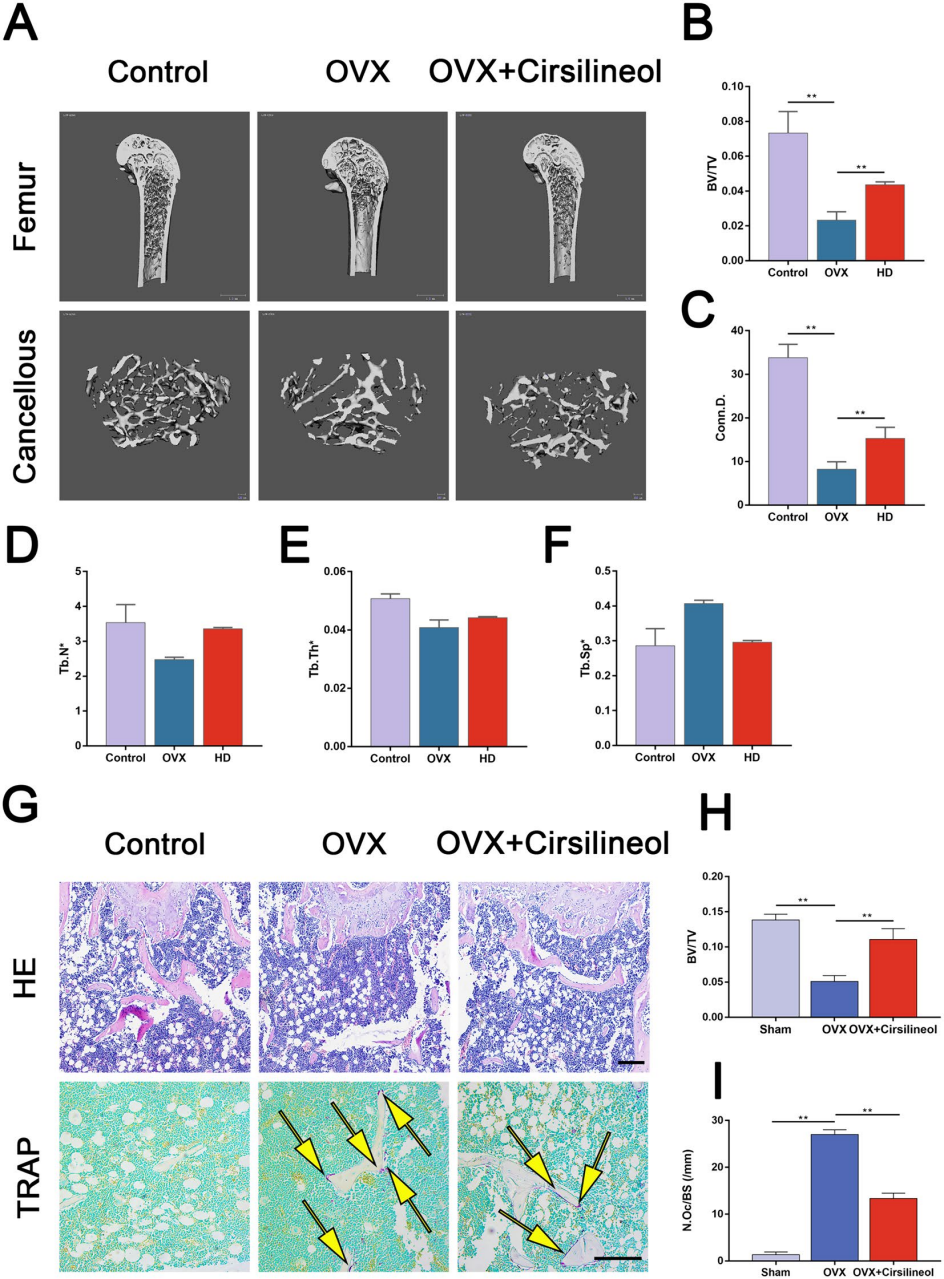
Cirsilineol ameliorates OVX-induced bone mass loss and osteoclast activation in vivo. **A**–**F** After the establishment of osteoporosis mice model, cirsilineol (20 mg/kg) was intraperitoneally injected into OVX mice for 1 month. Bilateral femurs were separated and photographed by micro-CT. The result verified that cirsilineol attenuated OVX-induced bone mass loss. The micromorphological quantification including BV/TV, Comn.D., Tb.N^*^, Tb.Th^*^ and Tb.Sp^*^ came to the same conclusion (n = 3). **G**–**I** The HE staining and TRAP staining results demonstrated that cirsilineol ameliorated bone mass loss and inhibited osteoclast activation at the histological level (n = 3). Scale bar (HE staining) = 200 μm; Scale bar (TRAP staining) = 100 μm. *P < 0.05, **P < 0.01 vs. the control group

In order to verify the possible therapeutic effect of cirsilineol in vivo*,* our study finally tested whether cirsilineol prevented bone volume loss and inhibited osteoclast action in OVX-induced osteoporosis mice. Thirty C57BL/6 female mice were randomly divided into six groups and treated with different dosage of cirsilineol as previously described. Zoledronate-treated group was served as a positive control group. After euthanized by overusing anesthetics, bilateral femurs were isolated and scanned by microcomputed tomography. An obvious bone loss occurred in the distal femurs of the OVX group mice, which indicated that successful osteoporosis mice models were established. Compared with the OVX group, cirsilineol treatment significantly increased bone volume in the femurs of osteoporosis animals. The therapeutic effect of cirsilineol at a concentration of 20 mg/kg is significantly better than that of 10 mg/kg, and there were no obvious differences between OVX + cirsilineol (20 mg/kg) group, OVX + cirsilineol (30 mg/kg) group and OVX + Zoledronate (0.1 mg/kg). All bone quality statistical results such as BV/TV, Conn.D, Tb.N, Tb.Th and TB.Sp amply proved the protective effect of cirsilineol (Additional file 1: Fig. S3A, B, Fig. 6A–F). Thus, we chose a dosage of 20 mg/kg for further study.

Afterwards, we investigated whether cirsilineol protected against bone loss in histological level. All specimens were decalcified for two weeks and then performed with histological staining, including H&E staining and TARP staining. The results indicated that cirsilineol suppressed bone mass loss in OVX mice. Furthermore, the number and area of osteoclasts in vivo were measured after performed with TRAP staining. And the results suggested that osteoclast formation and activity were enhanced in the OVX group and significantly decreased in the cirsilineol-treated group (Fig. 6G, H).

All these data confirmed that cirsilineol prevented OVX-induced bone mass loss and inhibited osteoclast activity in osteoporosis mice.

## Discussion

Osteoporosis, characterized by metabolic disorders of bone tissue, is a homeostasis disruption between bone formation and bone resorption [36]. Osteoporosis is often accompanied by bone deformities, pain and brittle fractures, resulting in a decline in life quality and a huge burden on public healthcare system. Considering the crucial role of osteoclast in osteolysis, regulation of hyperactive osteoclast activity seems to be a potential approach for treating disorders associated with abnormal bone metabolism.

In common pathological bone state such as rheumatoid arthritis and osteoporosis, the over-activated RANKL/RANK signaling cascade is the critical event resulting in osteoclast formation, survival and activation [37]. With M-CSF stimulation, RANK expression increases, and the binding of RANKL and RANK enhances the recruitment of TNF receptor-associated factor 6 (TRAF6). Subsequently, the TGF-b-activated kinase 1 (TAK1), which belongs to the downstream signal transduction factor of TRAF6, is phosphorylated and thus leads to the activation of the downstream signaling pathways, including the IKKα/β/Ikβα. Since Ikβα acts as an inhibitor to inactivate the downstream cascade, the phosphorylation of IKKα/β enhances the degradation of IκB, thus resulting in the activation of NF-κb and activated protein 1 (AP-1) [38]. Activated AP-1 and NF-κB facilitate the generation of NFATc1 and then increase osteoclast-related genes expression, thereby promoting osteoclast differentiation [39]. There are a plenty of receptors and transcriptional factors participating in osteoclast activation, and these molecules are considered to be mediated by NFATc1. Our research suggested that cirsilineol inhibited RANKL-induced NF-κb signaling pathways.

Significant promotion of osteoclast differentiation is previously reported when p38-MAPK axis is initiated [40]. RANKL-induced p38-MAPK activation regulates the expression of osteoclast-specific genes, which is involved in osteoclast formation. As for ERK, another subunit of MAPK super family, is activated with RANKL stimulation and regulates osteoclast formation and function [41]. In our study, we demonstrated that cirsilineol attenuated the phosphorylation of ERK and p38 upon RANKL stimulation.

Numerous drugs have been approved for treating osteoporosis. But in fact, the poor efficacy and severe side effects of these drugs limit the application for the clinical administration. Cirsilineol is one of the main high active components of vestita Wall and is a typical non-glycosylated flavonoid with anti-oxidant and anti-inflammatory activities. The chinese herb extract cirsilineol has been used to treat rheumatoid arthritis and dermatitis in traditional Chinese medicine for a long time. Cirsilineol significantly decreases the expression of proinflammatory mediators of LPS-stimulated macrophages at transcriptional and translational levels, thus improving the local inflammatory microenvironment [29]. Endogenously produced inflammatory factors generate strong oxydic free radicals that cause osteoclast activation and bone mass loss in osteoporosis [42]. Inhibition of various inflammatory factors levels may be one of the mechanisms in which cirsilineol exerted anti-osteoporosis effects. In our study, we confirmed that an anti-inflammatory nature compound cirsilineol reduced osteoclast formation in vitro and inhibited estrogen deficiency-induced bone loss by suppressing osteoclast activity in vivo.

Several limitations of our study still remain unsettled. Firstly, the exact mechanism of how cirsilineol inhibits osteoclast activation remains to be explored. Secondly, the intervention of cirsilineol on osteoblast differentiation and bone formation is still unknown. Lastly, the current delivery of cirsilineol is not suitable for the treatment of osteoporosis and still needs to be improved.

## Conclusion

In conclusion, our study suggested that cirsilineol attenuated osteoclast differentiation by impairing NFATc1 induction, mainly through the NF-κB/ ERK/p38 signaling pathways. Meanwhile, cirsilineol improved osteoporosis-induced bone mass loss via inhibiting osteoclast formation and function.

### Supplementary Information

**Additional file 1: Figure. S1** Cytotoxicity assay of cirsilineol to BMMs and MC3T3-E1 cells. **Figure. S2** Cirsilineol has no effect on osteoblast differentiation. **Figure. S3** Micro-CT assay.

## Data Availability

All the data and materials that support the findings of this study are included in the manuscript.
