# Supplementary material for: Cirsilineol inhibits RANKL-induced osteoclast activity and ovariectomy-induced bone loss via NF-κb/ERK/p38 signaling pathways
Source: Chin Med. 2024 May 14;19:69. doi: 10.1186/s13020-024-00938-6 (PMC11095037; doi:10.1186/s13020-024-00938-6)
Supplement: Supplementary file 1 — Additional file 1: Figure. S1 Cytotoxicity assay of cirsilineol to BMMs and MC3T3-E1 cells. Figure. S2 Cirsilineol has no effect on osteoblast differentiation. Figure. S3 Micro-CT assay. [file 13020_2024_938_MOESM1_ESM.docx]

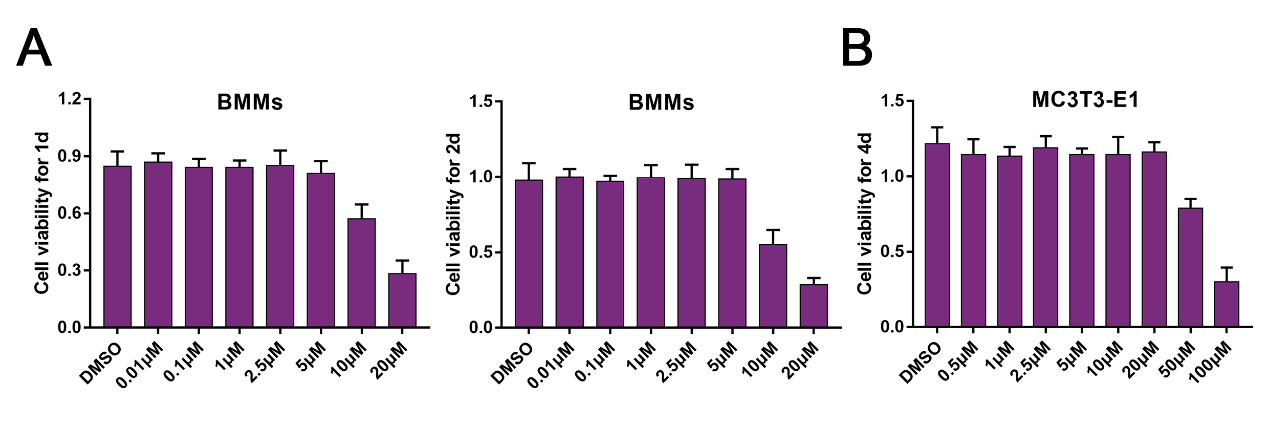


**Additional file 1: Fig. S1.** Cell viability assay. **(A)** Cell viability of BMMs after treated with different concentrations of cirsilineol **(**0, 0.01, 0.1, 1, 2.5, 5, 10, 20 μM**)** for 1 day and 2 days (n=4). **(B)** Cell viability of MC3T3-E1 cells after treated with different concentrations **(**0, 0.5, 1, 2.5, 5, 10, 20, 50, 100 μM**)** of cirsilineol for 4 days (n=4). *P < 0.05, **P < 0.01 vs. the control group.


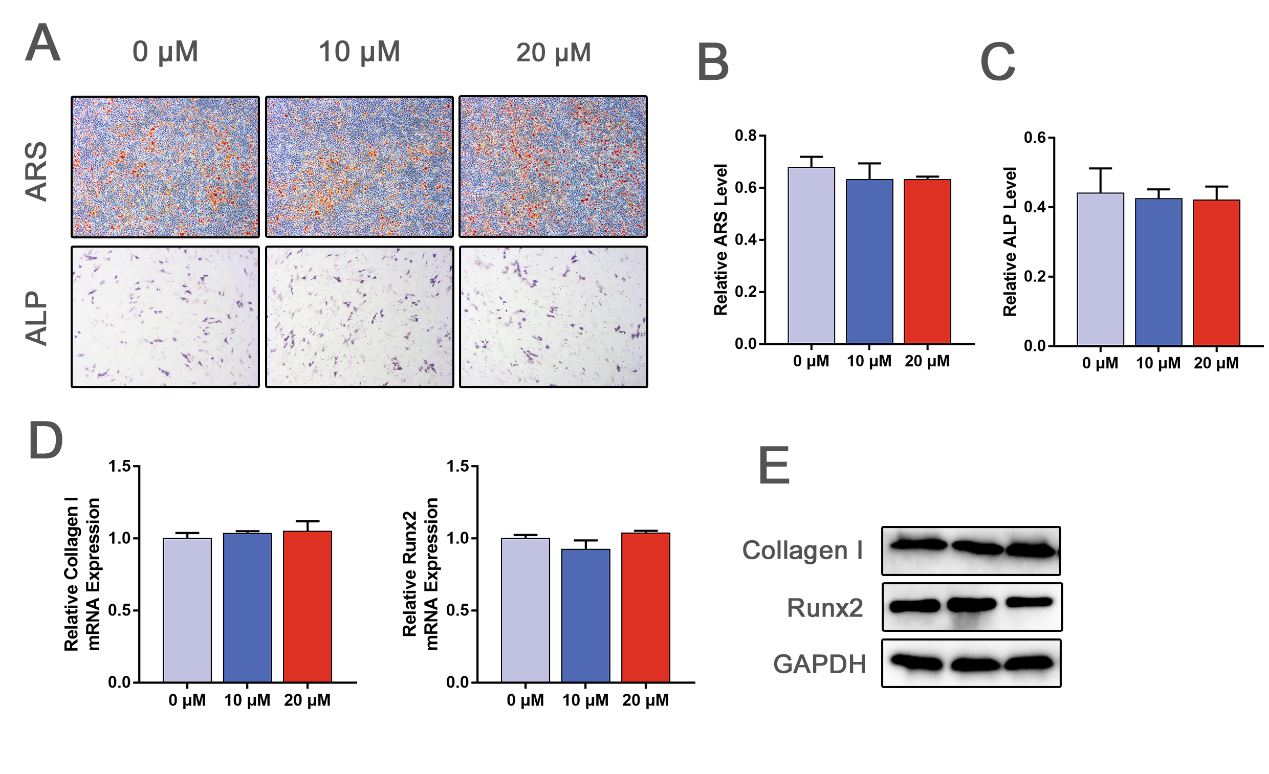


**Additional file 1: Fig. S2** Cirsilineol has no effect on osteoblast differentiation. (A) MC3T3-E1 cells were cultured with different concentrations of cirsilineol for 3 days and 14 days under osteogenic induction. ARS and ALP staining images showed no significant difference in osteogenic capacity. (B-C) Statistical results of ARS and ALP staining (n=3). (D) Detection of the mRNA expression of osteoblast-related genes including Collagen I and Runx2 by PCR (n=3). (E) Detection of the protein expression of osteoblast-related genes including Collagen I and Runx2 by Western Blot. *P < 0.05, **P < 0.01 vs. the control group.


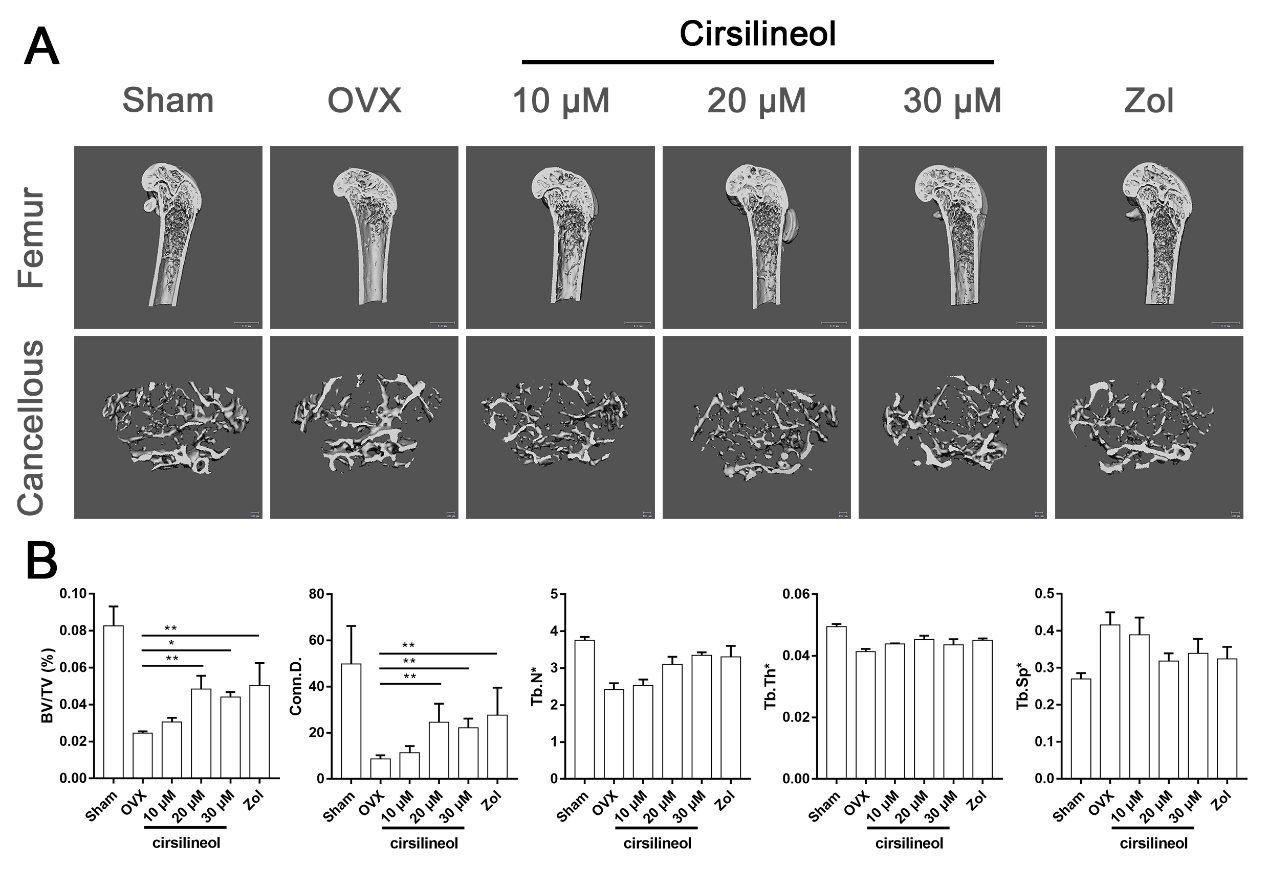


**Additional file 1: Fig. S3** Micro-CT assay. (A) Cirsilineol (10 mg/kg, 20mg/kg, 30mg/kg) and Zoledronate (0.1mg/kg) were administrated into OVX mice for 1 month. Detection of the microstructural changes of the femurs in each group by micro-CT. (B) Statistical results of the micromorphological quantification including BV/TV, Comn.D., Tb.N^*^, Tb.Th^*^ and Tb.Sp^*^ (n=3). Zol: Zoledronate. *P < 0.05, **P < 0.01 vs. the control group.
